# Sickness absence around contact with outpatient mental health care services – differences between migrants and non-migrants: a Norwegian register study

**DOI:** 10.1186/s12888-023-04874-x

**Published:** 2023-06-14

**Authors:** Melanie Straiton, Aart C. Liefbroer, Anna-Clara Hollander, Lars Johan Hauge

**Affiliations:** 1 Department of Mental Health and Suicide, Norwegian Institute of Public Health, PO Box 222, Skøyen, 0213 Oslo Norway; 2 Netherlands Interdisciplinary Demographic Institute, PO Box 11650, The Hague, 2502 AR The Netherlands; 3 Department of Epidemiology, University Medical Centre Groningen, University of Groningen, Groningen, The Netherlands; 4 Department of Sociology, Vrije Universiteit, Amsterdam, The Netherlands; 5 Epidemiology of Psychiatric Conditions, Substance Use and Social Environment, Department of Global Public Health, Karolinska Institute, Stockholm, 171 77 Sweden

**Keywords:** Sickness absence, Mental disorder, Mental health service use, Migrants, Refugees, Labour market marginalisation

## Abstract

**Background:**

Mental disorders are a leading cause of sickness absence. Some groups of migrants are at higher risk of both mental disorder and sickness absence. Yet, research on sickness absence in relation to mental disorders among migrants is limited. This study investigates differences in sickness absence in the twelve-month period around contact with outpatient mental health services between non-migrants and various migrant groups with different length of stays. It also considers whether these differences are similar for men and women.

**Methods:**

Using linked Norwegian register data, we followed 146,785 individuals, aged 18–66 years, who had attended outpatient mental health services and who had, or had recently had, a stable workforce attachment. The number of days of sickness absence was calculated for the 12-month period surrounding contact with outpatient mental health services. We applied logistic regression and zero-truncated negative binomial regression to assess differences in any sickness absence and number of days of absence between non-migrants and migrants, including refugees and non-refugees. We included interaction terms between migrant category and sex.

**Results:**

Refugee men and other migrant men from countries outside the European Economic Area (EEA) had a higher probability of any sickness absence in the period surrounding contact with outpatient mental health services than their non-migrant counterparts. Women from EEA countries with stays of less than 15 years had a lower probability than non-migrant women. Additionally, refugees, both men and women, with 6–14 years in Norway had more days of absence while EEA migrants had fewer days than their non-migrant counterparts.

**Conclusions:**

Refugee men and other non-EEA migrant men appear to have higher sickness absence than non-migrant men around the time of contact with services. This finding does not apply to women. Several probable reasons for this are discussed, though further research is required to understand why. Targeted strategies to reduce sickness absence and support the return to work for refugees and other non-EEA migrant men are needed. Barriers to timely help-seeking should also be addressed.

**Supplementary Information:**

The online version contains supplementary material available at 10.1186/s12888-023-04874-x.

## Background

Mental disorders are one of the leading causes of sickness absence (SA) and disability [1–3]. The duration of SA due to a mental disorder also appears to be longer than for many other conditions [4–6]. In several European countries, including Norway, a right to paid SA allows those with a mental disorder time to recover or cope without substantial loss of earnings. However, SA, particularly long SA due to mental disorders, can have unintended side effects such as prolonged or future SA, reduced future working hours and lower income [7–9]. It can also increase the risk of other types of labour market marginalisation such as future unemployment or disability pension [8–10]. It is noteworthy however, that labour market marginalisation can lead to, or contribute to the maintenance of, poorer mental health and increased risk of mental disorder [8, 11–13].

Migrants, and in particular, refugees appear to be at higher risk of labour market marginalisation such as unemployment and permanent disability pension [14–16]. Research on non-cause specific SA, however, is mixed and findings appear to vary by settlement country, migrant group, and different study designs [15, 17–22]. Research also indicates that migrants, and again, refugees in particular, may be at greater risk of developing some mental disorders [23–25]. This could, in turn, lead to higher rates of SA. A study on disability pension found that SA due to mental disorder may more often lead to permanent disability for migrants than non-migrants [10]. Thus, with an increasing migrant population in many European countries, knowing more about SA among migrants with mental disorders may be important for targeted prevention of future labour market marginalisation.

Existing research on migrants’ SA in relation to mental disorder comes predominately from Sweden. Among individuals diagnosed with post-traumatic stress disorder, the risk of a three-month or longer SA was 18% higher for refugees and 11% higher for non-refugee migrants compared with Swedish-born individuals [26]. Among young adults (19–30 years) diagnosed with common mental disorders (anxiety, depressive or stress disorders), migrants from Africa and Asia had lower likelihood of SA of least three months compared with their non-migrant counterparts [27]. Longer length of stay was associated with higher risk of SA for refugees and non-refugee migrants from Africa and Asia. Other migrant groups did not differ from the non-migrant population, though migrants from the European Union (EU) were excluded in both studies. Neither of these studies appeared to account for whether individuals were eligible for SA benefit (i.e. whether they were employed). In a study accounting for workforce attachment however, young adult non-migrants with any mental disorder had a higher risk of longer SA of at least three months than all studied migrant groups [28]. All of these studies have considered SA over a long period following a mental disorder diagnosis (6–7-year follow-up). There may be other contributing factors that could explain these differences given the long follow-up period, including new somatic health conditions, occupational differences, discrimination in the workplace or because previous absences increase the risk of future ones. Thus, it is unclear whether SA differs for migrants and non-migrants in the period *surrounding* the mental disorder diagnosis (or the use of mental health care services).

Apart from comparing SA of migrants and non-migrants with mental disorders in general, three additional comparisons are interesting. First, the reason for migration may be important. Refugees may be more prone to more complex mental health issues than other migrants as they often flee from traumatizing situations [23, 29]. This could result in more days of SA compared with other groups. Second, length of stay of migrants could matter, as health may deteriorate over time [30, 31]. SA may also increase over time as migrants adjust to the norms of the general population, though this could differ by migrant group [19]. Finally, patterns could be different for migrant men and women, given their different levels of labour market attachment [32, 33] and differences in their risk of mental disorder [25, 34].

### Current study setting and aims

In the current study, we investigate SA over a 12-month period among migrants and non-migrants who have, or have recently had, a workforce attachment and have attended outpatient mental health services (OPMH) in Norway. OPMH services are local specialised services where those with acute mental health problems or those requiring long-term follow-up can receive help. These services usually require a referral from a general practitioner (GP) and tend to be reserved for those with more serious or complex disorders. Most mild to moderate conditions are treated in primary care. All registered migrants with stays of over six months, and asylum seekers have the same rights to access health care services as the non-migrant population.

In Norway, SA benefit is paid for up to one year at 100% of one’s salary [35]. This is capped at six times the basic taxation amount (just under 70,000 euros in 2022). The SA benefit system is organised under the National Insurance Scheme, though the employer covers costs for the first 16 days of absence. Absences of more than 16 days per year need to be certified by a medical professional. Anyone who has been in work for at least 28 days prior to absence and is incapable of working or has reduced working capacity is entitled to this. Job seekers who have previously been in work are also entitled to SA benefit [36].

We investigate the following research questions:

1. Are the odds of any SA in the 12-month period surrounding contact with OPMH services different for migrants with different length of stays compared to non-migrants and does the strength of the relationship vary by sex?
2. Among those taking any SA, does the number of days of SA differ for migrants with different length of stays and does the strength of the relationship vary by sex?

## Method

### Study design and data sources

In this study, we used data from five national Norwegian databases and registries, which were linked at an individual level through a non-identifiable version of a personal number for the years 2006–2014. All Norwegian-born individuals and registered residents with at least six months of residence are assigned this personal number. The National Database for the Reimbursement of Health Expenses was used to identify individuals who had been in contact with OPMH services between 1st January 2007 and 31st March 2014. We obtained demographic information (e.g., sex, age, marital status, migrant category, year of arrival in Norway, reason for migration, country of origin) for these individuals from the National Population Registry. Educational level and income was extracted from the National Education Database and the National Income Tax Registry respectively. Finally, FD-Trygd, a database containing event history information relating to welfare benefits was used to extract information on SA benefit transfers.

### Study population

**Fig. 1:**
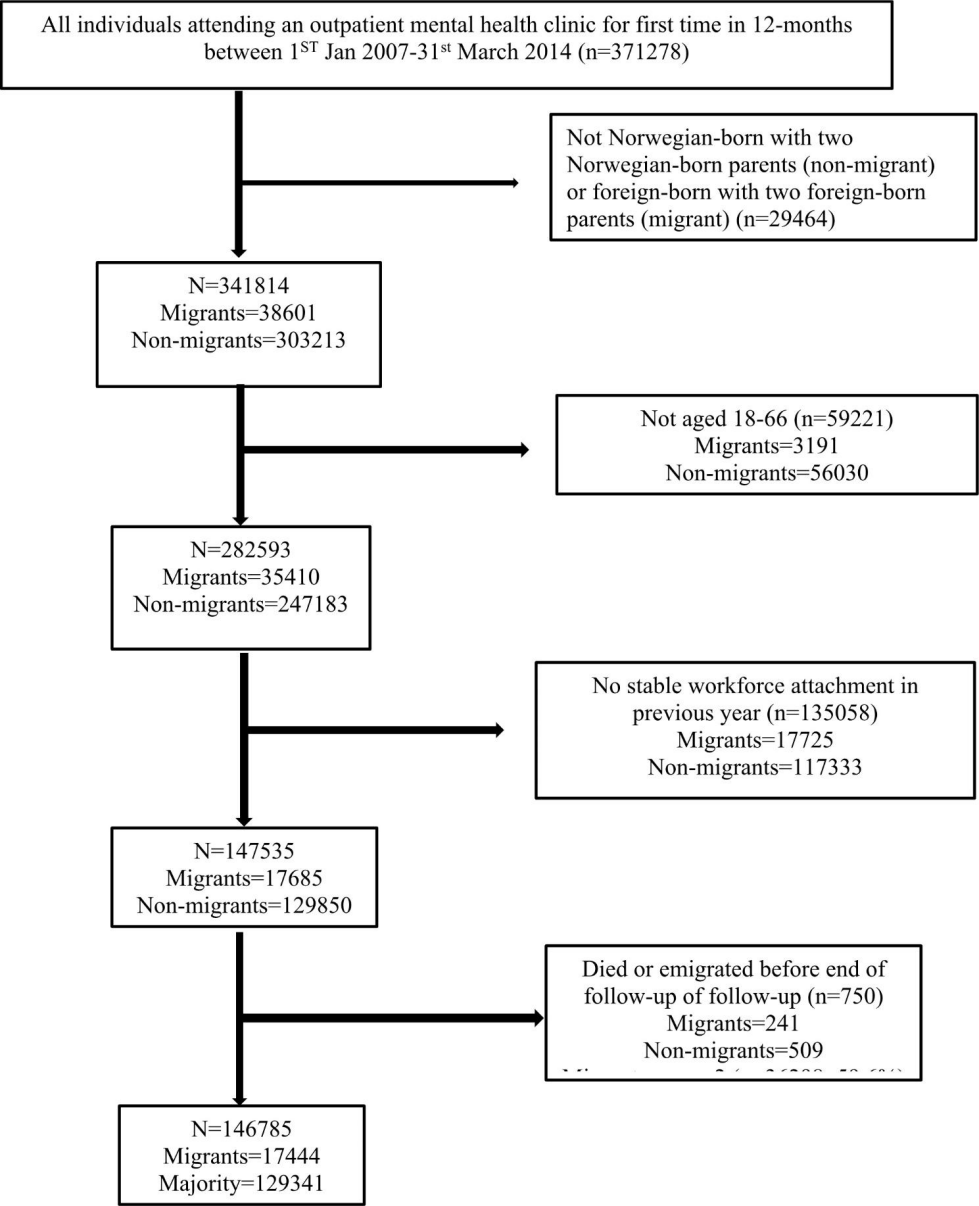
Sample inclusion criteria by migrant category

The study population included adults who had attended an OPMH clinic for the first time within one year (n = 371,278). The one-year washout period was applied in order to include new, rather than ongoing cases, of mental disorder. We were interested in comparing those born abroad with two foreign-born parents (migrants) to those born in Norway with two Norwegian-born parents (non-migrants) and therefore excluded other groups. We also limited the age range to 18–66 years as the age of retirement in Norway is 67 years. This allowed for a follow-up period following contact with OPMH services prior to retirement. In most cases, only employed individuals, or those who are actively job-seeking, are entitled to SA benefits. We therefore limited the sample to those who had earned over 1.5 times the basic taxation amount during the year prior to their first OPMH appointment. Use of this criterion may have excluded those who were newly entitled to SA benefits. It does, however, allow the inclusion of those who had recently been in work and therefore entitled to SA benefits [37]. At current 2022 levels, 1.5 basic taxation amount is 167,215 Norwegian kroner (NOK) (approximately 16,785 Euros) [38]. This equates to approximately 18 h per week for one year on a wage of 175 NOK (around 18 euros), which is the accepted basic wage for the service industry [39]. Thus, individuals who worked less than 18 h per week on this minimum wage are most likely excluded, while those working fewer hours on a higher salary are included. This criterion has previously been used as an indicator of a stable workforce attachment [40, 41]. Finally, we excluded those who died or emigrated during the follow-up period. This resulted in a sample population of 146,785. Migrants made up around 12% of the sample. See Fig. 1 for an overview of the sample inclusion criteria and the number of individuals excluded at each stage.

We observed all individuals for a 12-month period, starting 90 days prior to their first OPMH contact and ending 275 days after (and including) the first contact to see how many days of SA a person had. We included the prior 90 days because accessing OPMH services usually requires a referral from a GP and this can take some time. Thus, an individual is likely to have been struggling with a mental disorder in the period prior to OPMH contact, which in many cases could result in SA. Further, the GP is usually responsible for providing a medical certificate for SA. Initial analyses of the data confirmed that the overwhelming majority of those who experienced SA following contact with OPMH were already receiving SA benefit prior to their first OPMH contact. This supports our assumption that these two periods should be seen together rather than separately. In this way, we capture SA associated with mental disorder for those attending OPMH services, rather than only SA following contact with OPMH services.

### Variables

#### Outcomes

We included two outcome variables based on the SA data. Any SA in 365-day period surrounding the consultation (yes/no) and total number of days of SA in this period. Although SA can be taken on a part-time basis, we only counted the total number of days individuals received benefits (adding on the 16 days to reflect the total period of absence), rather than the percentage of benefits they were receiving.

#### Exposures

Migrant category:

- Non-migrants (Norwegian-born individuals with two Norwegian-born parents).
- Migrants (foreign-born abroad with two foreign-born parents).

Migrants were grouped into three categories based on reason for migration and country of birth:

- refugees (those given residency based on a recognised need for protection),
- non-refugee migrants from countries within the European Economic Area (EEA) (EEA other migrants).
- non-refugee migrants from countries outside of the EEA (non-EEA other migrants).

See additional file 1 for more details on the main countries represented in each category.

Length of stay: This was calculated based on year of OPMH consultation minus year of migration and then grouped as:

- short (≤ 5 years).
- medium (6–14 years).
- long (15 + years) lengths of stay in Norway.

Migrant stay: To examine if the link between SA and migrant category varied by length of stay, we used a combined categorical variable with the following categories:

- Non-migrants,
- Refugees, short stay.
- Refugees medium stay.
- Refugees, long stay.
- EEA other migrant, short stay.
- EEA other migrants, medium stay.
- EEA other migrants, long stay.
- non-EEA other migrants, short stay.
- non-EEA other migrants, medium stay.
- non-EEA other migrants, long stay.

#### Covariates

Sex: Man / Woman.

Age group: Age was calculated based on year of OPMH consultation minus year of birth and then grouped as:

- 18–29 years.
- 30–39 years.
- 40–49 years.
- 50–66 years.

We did not differentiate between those aged 50–59 and 60 + due to the relatively small number of migrants aged over 60 years in the sample (n = 319).

Marital status: Marital status in the year of OPMH consultation was categorised as:

- married.
- never married.
- separated, divorced, widowed.

Educational level: Highest level of education in the year of OPMH consultation, categorised as:

- <=compulsory education/unknown.
- upper secondary.
- lower college/university education.
- upper college/university education (e.g. Master’s degree or equivalent or higher).

Income level: Income before taxation was measured in the year prior to OPMH consultation and grouped into three levels:

- low income (1.5–3 times the basic taxation amount).
- middle income (3–6 times the basic taxation amount).
- higher income (> 6 times the basic taxation amount).

At 2022 levels, the basic taxation amount was 111 477 NOK (around 11 525 euros). Thus, those with higher income earned at least 69 153 euros in the year prior to OPMH consultation.

Number of OPMH consultations: The number of consultations is a proxy indication of severity or complexity of the disorder. We followed individuals for 275 days from their first OPMH consultation and grouped the total number of OPMH consultations in this time period (including the first) as:

- ≤ 5 contacts.
- 6–10 contacts.
- 11–15 contacts.
- > 15 contacts.

Year of study inclusion: Since SA practices may change over time, we also controlled for year of study inclusion (year of OPMH appointment) (2007–2014).

### Analysis

Descriptive statistics by migrant category were calculated to describe the sample according to demographics and other covariates. We also estimated the prevalence of SA and the mean and median number of days of absence by migrant category.

For the first research question, we ran logistic regression analyses for the exposure variable (migrant stay) to determine if the odds of SA differed for the different migrant groups with different length of stays compared with non-migrants. We also ran logistic regression analyses for each of the covariates separately to see if and how they related to any SA. We then ran a fully adjusted model with all variables to assess whether the odds of SA differed for the different migrant groups with different length of stays compared with non-migrants when controlling for all covariates. To the fully adjusted model, we then added in an interaction term between sex and migrant stay to see if any differences in odds of SA between the different migrant groups and non-migrants was applicable to both men and women. Finally, based on this model, we calculated marginal predicted probabilities of SA for the different migrant groups by sex to improve the readability of the results. For the second research question, we excluded individuals who had no SA in the 12-month period surrounding the OPMH contact. We then ran zero-truncated negative binomial regression analyses to determine if there were differences between the different migrant groups with different length of stays and non-migrants in the number of days of SA. Again, we also ran analyses for each of the covariates separately before including a fully-adjusted model with all variables. Finally, we included an interaction term between sex and migrant stay.

## Results

### The sample

Of the included 146,785 individuals, 55% were women. Of the 17,444 migrants in the sample, 21% were refugees, 38% EEA other migrants and 41% non-EEA other migrants. Table 1 provides an overview of the characteristics of the sample by migrant category.

**Table 1:** Overview of demographic variables and sickness absence by migrant category

		Migrants			
	Non-migrants	Refugees	EEA countries - other	Non-EEA countries - other	Total
Total	129,341 (88.12%)	3710 (2.53%)	6652 (4.53%)	7082 (4.82%)	146,785 (100.00%)
Men	57,749 (44.65%)	2188 (58.98%)	3050 (45.85%)	3027 (42.74%)	66,014 (44.97%)
Women	71,592 (55.35%)	1522 (41.02%)	3602 (54.15%)	4055 (57.26%)	80,771 (55.03%)
Age group					
18–29 years	32,354 (25.01%)	911 (24.56%)	1209 (18.17%)	1431 (20.21%)	35,905 (24.46%)
30–39 years	36,831 (28.48%)	1234 (33.26%)	2407 (36.18%)	2636 (37.22%)	43,108 (29.37%)
40–49 years	32,504 (25.13%)	1083 (29.19%)	1801 (27.07%)	2004 (28.30%)	37,392 (25.47%)
50–66 years	27,652 (21.38%)	482 (12.99%)	1235 (18.57%)	1001 (14.28%)	30,380 (20.70%)
Marital status					
Married	41,948 (32.42%)	1988 (53.58%)	2595 (39.01%)	4035 (56.98%)	50,566 (34.45%)
Never married	65,806 (50.88%)	1115 (30.05%)	2952 (44.38%)	1429 (20.18%)	71,302 (48.58%)
Separated, divorced, widowed	21,587 (16.69%)	607 (16.36%)	1105 (16.61%)	1618 (22.85%)	24,917 (16.98%)
Educational level					
≤ lower secondary/unknown	44,611 (34.49%)	1651 (44.50%)	2578 (38.76%)	3400 (48.01%)	52,240 (35.59%)
Upper secondary	46,591 (36.02%)	1205 (32.48%)	1496 (22.49%)	1693 (23.91%)	50,985 (34.73%)
Lower college/university	31,357 (24.24%)	664 (17.90%)	1722 (25.89%)	1372 (19.37%)	35,115 (23.92%)
Upper college/university	6782 (5.24%)	190 (5.12%)	856 (12.87%)	617 (8.71%)	8445 (5.75%)
Income level					
Low	30,095 (23.27%)	985 (26.55%)	1380 (20.75%)	1881 (26.56%)	34,341 (23.40%)
Middle	72,315 (55.91%)	2316 (62.43%)	3857 (57.98%)	4066 (57.41%)	82,554 (56.24%)
Higher	26,931 (20.82%)	409 (11.02%)	1415 (21.27%)	1135 (16.03%)	29,890 (20.36%)
Length of stay					
Short		421 (11.35%)	2527 (37.99%)	1075 (15.18%)	4023 (23.06%)
Medium		1935 (52.16%)	2165 (32.55%)	2471 (34.89%)	6571 (37.67%)
Long		1354 (36.50%)	1960 (29.46%)	3536 (49.93%)	6850 (39.27%)
OPMH consultations					
≤ 5	57,959 (44.81%)	1718 (46.31%)	2961 (44.51%)	3305 (46.67%)	65,943 (44.92%)
6–10	30,640 (23.69%)	822 (22.16%)	1512 (22.73%)	1596 (22.54%)	34,570 (23.55%)
10–15	19,508 (15.08%)	576 (15.53%)	1019 (15.32%)	1053 (14.87%)	22,156 (15.09%)
> 15	21,234 (16.42%)	594 (16.01%)	1160 (17.44%)	1128 (15.93%)	24,116 (16.43%)
Median year of study inclusion	2010	2010	2011	2010	2010
Any sickness absence	83,503 (64.56%)	2525 (68.06%)	4208 (63.26%)	4769 (67.34%)	95,005 (64.72%)
Mean (sd) days of absence*	190.59 (108.21)	200.08 (110.54)	190.42 (109.24)	191.33 (109.45)	190.88 (108.39)
Median days of absence*	186	203	187	188	187

*reported for among those with any sickness absence

Overall, around 65% of individuals had any SA in the period between 90 days before and 275 days after the OPMH consultation. SA differed significantly across migrant category (*X*^2^[3] = 47.07, p < 0.001), being highest among refugees (68%) and lowest among EEA other migrants (63%). The mean number of days among those who had any SA in the period surrounding contact with OPMH services was 191 and the median was 187. The mean number of days differed significantly by migrant category (F[2] = 6.31, p < 0.001). Refugees had the highest mean number of days of SA while the other groups had a similar mean.

### Any SA around the time of OPMH consultation

Table 2 shows the unadjusted and adjusted odds of having any SA for each of the covariates. In the unadjusted analyses, refugees and migrants with short stays had lower odds of SA than non-migrants, though this was only statistically significant for EEA other migrants. Both refugees and non-EEA other migrants who had medium or long stays in Norway had statistically higher odds of SA than non-migrants, while EEA other migrants had similar odds to non-migrants.

**Table 2:** Odds ratios (OR) and 95% confidence intervals (CI) for any sickness absence

	Unadjusted OR (95% CI)	Fully adjusted OR (95% CI)
Migrant stay		
Non-migrants	1	1
Refugees, short stay	0.85 (0.70–1.04)	1.15 (0.94–1.41)
Refugees, medium stay	1.22 (1.11–1.35)***	1.28 (1.15–1.41)***
Refugees, long stay	1.22 (1.08–1.37)**	1.20 (1.06–1.35)**
EEA other migrants, short stay	0.84 (0.77–0.91)***	0.92 (0.84–0.99)*
EEA other migrants, medium stay	0.97 (0.89–1.06)	0.90 (0.83–0.99)*
EEA other migrants, long stay	1.07 (0.98–1.18)	0.98 (0.89–1.08)
non-EEA other migrants, short stay	0.89 (0.79–1.01)	0.99 (0.87–1.13)
non-EEA other migrants, medium stay	1.15 (1.06–1.26)**	1.12 (1.02–1.22)*
non-EEA other migrants, long stay	1.20 (1.12–1.29)***	1.13 (1.05–1.22)**
Sex		
Men	1	1
Women	1.41 (1.38–1.44)***	1.54 (1.50–1.58)***
Age group		
18–29 years	1	1
30–39 years	1.53 (1.48–1.57)***	1.28 (1.24–1.32)***
40–49 years	1.68 (1.63–1.74)***	1.36 (1.31–1.41)***
50–66 years	1.58 (1.52–1.62)***	1.31 (1.26–1.36)***
Marital status		
Married	1	1
Never married	0.73 (0.72–0.75)***	0.89 (0.87–0.92)***
Separated, divorced, widowed	1.03 (0.99–1.06)	0.97 (0.94-1.00)
Educational level		
≤ lower secondary/ unknown	1	1
Upper secondary	0.91 (0.88–0.93)***	0.83 (0.81–0.85)***
Lower college/university	1.03 (0.98–1.06)	0.76 (0.74–0.78)***
Upper college/university	0.79 (0.75–0.83)***	0.59 (0.56–0.62)***
Income level		
Low	1	1
Middle	2.28 (2.23–2.34)***	2.30 (2.24–2.36)***
Higher	1.88 (1.81–1.94)***	2.16 (2.09–2.25)***
Number of OPMH contacts		
≤ 5 contacts	1	1
6–10 contacts	1.24 (1.20–1.27)***	1.25 (1.21–1.28)***
10–15 contacts	1.48 (1.43–1.53)***	1.48 (1.44–1.53)***
> 15 contacts	1.90 (1.84–1.96)***	1.92 (1.85–1.98)***
Year of first OPMH contact	0.98 (0.98–0.99)***	0.97 (0.97–0.98)***
Number of observations = 146 785		

*p < 0.05, **p < 0.01, ***p < 0.001

In the fully adjusted model, all groups of refugees had higher odds of SA than non-migrants, though estimates were only statistically significant for those with medium and long stays in Norway. EEA other migrants with short stays had significantly lower odds than non-migrants, while non-EEA other migrants with medium and long stays had higher odds.

Overall, women had more than 50% higher odds of any SA compared with men. The odds of SA increased with age group, peaking between the ages of 40–49 years. Never married individuals had lower odds of SA than married individuals. SA appeared to decrease with increasing educational level, but the relationship was more complex for income. Those with a middle level income had the highest odds, followed by those with the highest income level. An increasing number of OPMH consultations was associated with increased odds of SA. Finally, the year of study inclusion was significantly negatively associated with the odds of SA, indicating that SA decreased slightly over time.

**Fig. 2:**
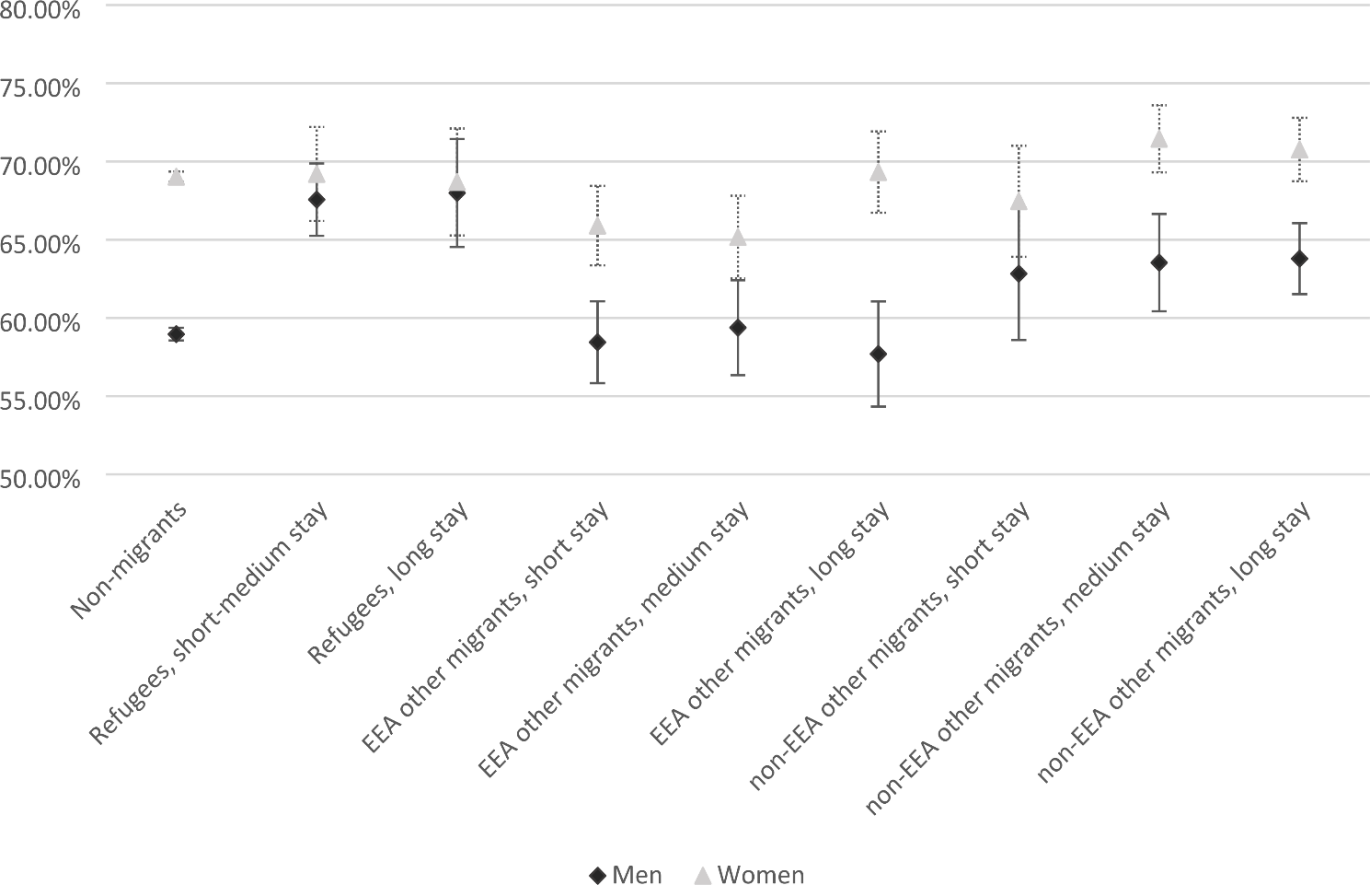
Marginal predicted probability (%) of any sickness absence by migrant stay and gender

To see if the differences in odds of SA across migrant stay applied to both men and women, we included an interaction term between migrant stay and sex in the model. Due to the small number of refugees, especially women (n = 105), with short stays in Norway, we combined refugees with short and medium stays (refugees < 15 years). The interaction term was significant for several groups (see additional file 2). We calculated marginal predicted probabilities for migrant stay and sex at the means of all other covariates and plotted the results as percentages. Figure 2 shows that for men, refugees had significantly higher probability of SA than their non-migrant counterparts (around 8% difference). There was no overall difference in the marginal probability of SA between non-migrants and EEA other migrants, regardless of length of stay. In contrast, non-EEA other migrants with medium and long stays in Norway had significantly higher probability of SA than non-migrants (around 4% difference). For women, the picture was different. EEA migrants short and medium stays had significantly lower probability of SA than their non-migrant counterparts (3–4% difference) but there was no significant difference for any other group, including refugees. The figure also suggests that the observed sex difference in the non-migrant population (elevated for women), is smaller in most migrant groups, except for EEA migrants with long stays in Norway.

### Days of SA around the time of OPMH consultation

Table 3 shows the unadjusted and adjusted incidence rate ratios (IRR) for the number of SA days for each of the covariates. Analyses only included individuals with at least one day of SA (n = 95,005). In the unadjusted analyses, refugees with medium and long stays in Norway had a significantly higher IRR, indicating that the number of SA days was approximately 6% higher than for non-migrants.

**Table 3:** Incidence rate ratio (IRR) and 95% confidence intervals (CI) for days of sickness absence

	Unadjusted IRR (95% CI)	Fully-adjusted IRR (95% CI)
Non-migrants	1	1
Refugees, short stay	0.97 (0.88–1.04)	0.99 (0.91–1.08)
Refugees, medium stay	1.06 (1.02–1.10)**	1.06 (1.02–1.10)**
Refugees, long stay	1.06 (1.01–1.11)**	1.03 (0.99–1.07)
EEA other migrants, short stay	0.97 (0.94-1.00)	0.96 (0.93–0.99)*
EEA other migrants, medium stay	1.00 (0.97–1.04)	0.98 (0.94–1.01)
EEA other migrants, long stay	1.03 (0.99–1.07)	1.00 (0.97–1.04)
non-EEA other migrants, short stay	0.96 (0.91–1.01)	0.97 (092-1.02)
non-EEA other migrants, medium stay	0.99 (0.96–1.03)	0.99 (0.96–1.02)
non-EEA other migrants, long stay	1.02 (0.99–1.05)	1.01 (0.98–1.04)
Sex		
Men	1	1
Women	1.01 (1.00-1.02)**	1.00 (0.99–1.01)
Age group		
18–29 years	1	1
30–39 years	1.08 (1.07–1.10)***	1.08 (1.07–1.10)***
40–49 years	1.10 (1.09–1.17)***	1.11 (1.09–1.12)***
50–66 years	1.12 (1.11–1.14)***	1.14 (1.12–1.16)***
Marital status		
Married	1	1
Never married	0.98 (0.95–0.97)***	0.99 (0.99-1.00)
Separated, divorced, widowed	1.00 (0.99–1.02)	0.99 (0.98–1.01)
Educational level		
≤ lower secondary/ unknown	1	1
Upper secondary	0.96 (0.95–0.97)***	0.95 (0.94–0.96)***
Lower college/university	0.99 (0.98–0.998)*	0.95 (0.94–0.96)***
Upper college/university	0.96 (0.94–0.98)***	0.93 (0.91–0.95)***
Income level		
Low	1	1
Middle	1.06 (1.04–1.07)***	1.03 (1.02–1.05)***
High	1.02 (1.01–1.04)**	1.00 (0.98–1.01)
Number of OPMH contacts		
≤ 5 contacts	1	1
6–10 contacts	1.14 (1.13–1.16)***	1.15 (1.13–1.16)***
10–15 contacts	1.25 (1.23–1.26)***	1.25 (1.23–1.26)***
> 15 contacts	1.34 (1.32–1.35)***	1.34 (1.33–1.36)***
Year of first OPMH contact	1.02 (1.02–1.02)***	1.02 (1.02–1.02)***
Number of observations = 95 005		

*p < 0.05. **p < 0.01. ***p < 0.001

In the fully adjusted model, refugees with medium stays had a significantly higher IRR, indicating that they had approximately 6% more days of SA than non-migrants. EEA other migrants with short stays in Norway had a slightly but significantly lower IRR, indicating that the number of SA days was approximately 4% lower than for non-migrants. There were no other statistically significant differences by migrant stay. SA days increased with increasing age group. Sex and marital status however, was not related to the incident rate ratio. Educational level was negatively associated with SA days while those with middle level income had more days of absence than those with a low income. The number of days of SA was associated with a higher number of OPMH contacts and with increasing year of study inclusion.

Again, due to the small number of refugees with shorter stays in the analysis (men = 190, women = 66), we combined those with short and medium stays (refugees < 15 years) when including the interaction term. The interaction term was not significant for any of the groups, indicating that relationship between migrant category and number of days of SA was similar for men and women.

## Discussion

We had two primary aims in this study. The first was to see if the odds of any SA and the number of days of SA in the 12-month period surrounding contact with OPMH services differed for migrants with varying length of stays compared to non-migrants. The second was to investigate if the relationship differed for men and women. We found several interesting contrasts between non-migrants and each of the migrant groups. Differences were not consistent for men and women and varied by length of stay. We discuss these specific differences further below after commenting on general findings.

Overall, we found 65% of individuals who attended OPMH services had any SA in the 12-month period surrounding this contact. Moreover, absences were long, with the median being around six months. Thus, SA in the period surrounding OPMH service use is substantial, attesting to the seriousness and complexity of the mental disorders among those attending services. In line with some, but not all, research on SA related to mental disorder, women were overall more likely to have any SA than men [42, 43]. Absences were, however, not significantly longer for women, confirming a previous study [6]. Further, within each migrant group, the overall pattern was that SA was lower for migrants with shorter stays than for migrants with medium or longer stays. This has been found in previous research on non-cause specific SA [19, 27]. Such an increase could indicate an adaption to SA norms in Norway or a decline in health over time [19].

A key finding of this study is that refugee men who had attended OPMH services had a higher probability of SA than non-migrant men, and that refugees (both men and women) also had around 7% more days of SA than non-migrants. Refugees appear to be at higher risk of more serious or complex mental disorders than non-migrants [25] and research suggests that more severe disorders are associated with higher risk of long-term SA [44]. Thus, this may explain why refugees have more days of SA than non-migrants. We also found that non-refugee men from non-EEA countries with medium and long stays in Norway have higher probability of SA than their non-migrant counterparts. One explanation for these findings relates to barriers to care. Barriers are well-documented among migrants [45–47] which, at best, can lead to delayed help-seeking. This may particularly be the case for refugees and other migrants from countries where mental health problems are particularly stigmatised, such as in many parts of Asia and Africa [47–49]. Thus, the mental disorder could be further progressed or more severe around the time of OPMH contact for refugees and non-EEA other migrants, explaining the higher probability of SA for these groups. There may therefore be a need for more timely access to mental health services among these groups. Another explanation for the high SA among refugee and other migrant men from non-EU countries could be that they experience discrimination in the workplace [50] which, coupled with discrimination for having a mental disorder, could increase the risk of SA [51]. A third explanation could be that refugees and non-EEA other migrants often occupy lower paid and more physically demanding jobs that allow for less control over the working day than non-migrants [52]. The less flexible nature of their work may make it harder to combine work with a mental disorder, explaining the higher level of SA. Previous research suggests that work-related factors, including control, may be important predictors of SA among those with mental disorders [7, 53]. Finally, more physically demanding jobs may increase the risk of physical injury or health problems, also increasing the risk of SA [54].

Yet, we did not observe a higher level of SA for refugee or non-EEA other women compared with non-migrant women. It is possible that this is due to predominant gender roles and differences in labour market attachment between men and women in these groups. A far lower proportion of women from refugee-generating countries and other non-EEA countries are employed than both non-migrant women and EEA other migrant women [33]. Thus, there may be greater selection of refugee and other non-EEA women with the poorest mental health out of the workforce. If so, women with the most serious disorders in these groups would have been excluded from our study. Refugee men and other non-EEA migrant men attending OPMH services, in contrast, may have more responsibility for providing for their families and continue to stay in the workforce despite their difficulties, leading to lower health selection among men, but higher SA. This may especially apply to men during their first five years of residency in Norway, where many men, particularly refugees, may be aiming to bring their families to safety in Norway. To do this, proof of being able to support family members through their own earnings is required [55]. This could also explain why we observed a higher number of days of SA for refugees with medium stays and not for those with shorter stays.

In addition, working women from refugee-generating countries and other non-EEA countries, more often work part-time than non-migrant women [56]. Not having the demand to be at work every day may allow for a better work-life balance and more time for recovery, making it easier to combine work with a mental disorder. Indeed, Swedish research suggests that part-time workers have lower odds of SA [57]. This explanation may also be supported by our finding that those with the lowest income had the lowest odds of SA, since lower income may reflect greater likelihood of working fewer hours. However, it is also possible that those with poorer mental health select themselves into, or only obtain, low paid, part-time work. Such individuals would be excluded from this study. We may therefore be underestimating the relationship between mental disorder and SA for the whole working population.

Another important finding was that non-refugee women from the EEA with short and medium stays in Norway had a lower probability of SA in the period surrounding contact with OPMH services than non-migrant women. EEA migrants tend to have higher education and better income than other migrants, and in this sample, than their non-migrant counterparts. These factors may relate to better mental health literacy, which is important for timely help seeking [58]. If they sought help at an earlier stage, they may have had less need for SA. Given their higher education and income, it is also possible that EEA women are more often in more flexible jobs that can be adapted to the individual’s needs, allowing those with a mental disorder to remain in work. This is supported by a previous study finding that migrant women from Western and Eastern European EU countries did not suffer from the same loss of income following treatment in OPMH services as other groups of migrant and non-migrant women [59].

Additionally, it is possible that many EEA migrants are in temporary positions [60]. Since SA may make it harder to obtain new employment [61], some may not take SA for fear of not gaining further employment. This argument could apply especially to women with shorter stays, who do not yet have permanent residency (around five years) or citizenship (from seven years). Previous research also suggests that migrants’ SA increases with length of stay – perhaps because migrants acclimatise to the social norms in the country of settlement [19]. Why this finding only applied to women however, and not to men from EEA countries is unclear. We did find though, that among those who had any SA, EEA other migrants with short stays had around 4% fewer days of absence than their non-migrant counterparts, regardless of sex.

Overall, this study attests to the complex ways in which sex, migrant status, and length of stay interact in shaping patterns of SA among migrants with mental disorder in Norway. It feeds into a larger literature that focusses on labour market marginalisation differences between migrants and non-migrants [10, 14, 15, 26, 28, 59]. An intersectionality approach where migrant sex, migrant group, and migrant length of stay are all considered is needed; both in our scientific approach and in working on prevention and interventions for SA and other forms of labour market marginalisation among migrants.

### Strength and limitations

The design of this study has both strengths and weaknesses. The use of register data allowed us to identify all individuals within a specific time frame who have (a) been in contact with OPMH services and (b) had a SA in the 12-month period surrounding this. SA data is highly accurate since this data is used in benefit transfers, increasing the validity of our study. Although we did not have diagnosis information, we were able to control for number of appointments with OPMH services which gives some indication of the severity of the mental disorder. As expected, there was a strong association between the number of OPMH consultations and SA.

Another positive aspect is our investigation of SA around the time of OPMH use. Previous studies on SA and mental disorder among migrants have mostly considered future SA, with a follow-up of several years after diagnosis [26–28]. However, we did not have access to information on the main cause of SA. Some of the SA in this study could relate to physical health conditions. Yet, mental disorders have a significant impact on a person’s functioning and health and there is high comorbidity with many chronic health problems such as musculoskeletal pain, cancer and cardiovascular diseases [62]. Thus, even if the main cause of SA is another condition, mental disorders significant enough to reach the attention of OPMH services are likely to play a role in long-term SA. It should also be emphasised that we do not capture absences of less than 16 days (where SA benefits are only covered by the employer). Additionally, we did not account for whether individuals had part or full-time SA, just the total number of days they were receiving SA benefit. As such, it is not known if the groups at higher risk of SA are overall more costly to society.

We only included individuals who had had contact with OPMH services. This ensured that all individuals in the study had mental health difficulties serious enough to be referred to specialist services and that everyone had overcome barriers to accessing them. This means however, that our findings are not generalisable to those with mental disorders who have not accessed OPMH services. Further, focusing only on users of OPMH services may have resulted in a somewhat more selective migrant sample. We know that barriers to care among migrants are numerous [45–47] and it is possible that those who have accessed OPMH care are more resourceful than migrants with mental disorders who have not accessed care [63, 64].

Another limitation is the lack of information on work-related factors. We were unable to differentiate between part-time and full-time work, private and public sector and the type of industry or position within an industry. These may be important predictors of SA [7, 53]. Given differences in the types of positions migrants, particularly those with lower education, tend to occupy, not controlling for this could potentially over-estimate the odds of SA among migrants compared with non-migrants. By including diagnosis information and work-related factors in future studies, we may also be able to determine the reasons for the observed differences in SA across the groups. Future studies could also investigate the role of age of migration because this may influence migrants’ knowledge of and ability to access health and welfare services [27]. Understanding why there are differences between migrants and non-migrants’ SA in the period surrounding use of OPMH services is important when designing targeted strategies to reduce the risk of labour market marginalisation among migrants with mental disorders. Additionally, improved employer support and return to work schemes following mental disorder may be particularly important for refugees who tend to have more days of SA in the period surrounding OPMH service use.

## Conclusion

Our findings show some differences in SA in the period surrounding contact with OPMH services across migrant category and stay compared to non-migrants. The pattern differed for men and women for any SA but not for the number of days of SA. EEA-women had lower probability of any SA than non-migrant women, though this difference faded with increasing length of stay. In contrast, refugee men and other migrant men from non-EEA countries had a higher probability of SA than non-refugee men. Refugees with medium stays also took around 7% more days than their non-migrant counterparts. This could be because refugees are at higher risk of more serious and complex disorders. Both refugee men and men from non-EEA countries may also experience significant barriers to accessing care. This could mean that mental disorders are more progressed by the time care is accessed, resulting in a greater need for, and longer periods of, SA. Since long-term SA has been associated with future labour market marginalisation, efforts should be made to minimise the risk of future labour market marginalisation for these groups, as well as individuals with more serious and complex disorders. Reducing barriers to care and encouraging prompt help-seeking may also be important for migrants.

## Electronic supplementary material

Below is the link to the electronic supplementary material.

Additional file 1: Countries represented in each of the migrant categories

Additional file 2: Interaction analyses

## Data Availability

The datasets generated and analysed for the current study are not publicly available for data protection reasons. However, the data that support the findings of this study may be available from Statistics Norway and HELFO if ethical approval is granted.

## List of abbreviations

SA: Sickness absence
EU: European Union
OPMH: Outpatient mental health
EEA: European Economic Area
GP: General practitioner
IRR: Incidence rate ratios
