# Supplementary material for: Sickness absence around contact with outpatient mental health care services – differences between migrants and non-migrants: a Norwegian register study
Source: BMC Psychiatry. 2023 Jun 14;23:428. doi: 10.1186/s12888-023-04874-x (PMC10265815; doi:10.1186/s12888-023-04874-x)
Supplement: Supplementary file 1 — Additional file 1: Countries represented in each of the migrant categories [file 12888_2023_4874_MOESM1_ESM.docx]

Additional file 1: Countries represented in each of the migrant categories

| Migrant category | Number of countries represented | Countries making up around 2/3 of sample |
| --- | --- | --- |
| Refugees | 93 | Bosnia Herzegovina (21%) Iraq (16%), Iran (13%), Kosovo (11%), Afghanistan (7%) |
| EEA other | 33^ | Sweden (27%), Poland (13%), Germany (11%), Denmark (10%), UK (7%) |
| Non-EEA other | 127 | Iran (9%), Turkey (9%), Pakistan (7%), Chile (6%), Russia (5%), Morocco (4%), USA (4%), Thailand (4%), Iraq (4%), Vietnam (4%), Sri Lanka (3%), The Philippines (3%), India (3%) |
| ^Includes all EEA countries and Greenland and Faro Islands since inhabitants have Danish citizenship and freedom of movement in the EU | | |
