# Supplementary material for: Sickness absence around contact with outpatient mental health care services – differences between migrants and non-migrants: a Norwegian register study
Source: BMC Psychiatry. 2023 Jun 14;23:428. doi: 10.1186/s12888-023-04874-x (PMC10265815; doi:10.1186/s12888-023-04874-x)
Supplement: Supplementary file 2 — Additional file 2: Interaction analyses [file 12888_2023_4874_MOESM2_ESM.docx]

Additional file 2: Interaction analyses

| **Odds ratios (OR) and 95% confidence intervals (CI) for any sickness absence^1^** | |
| --- | --- |
|  | Fully adjusted OR (95% CI) |
| Migrant stay |  |
| Non-migrants | 1 |
| Refugees, short-medium stay | 1.43 (1.27-1.60)*** |
| Refugees, long stay | 1.47 (1.25-1.74)*** |
| EEA other migrants, short stay | 0.98 (0.87-1.09) |
| EEA other migrants, medium stay | 1.01 (0.88-1.15) |
| EEA other migrants, long stay | 0.94 (0.81-1.09) |
| non-EEA other migrants, short stay | 1.13 (0.94-1.38) |
| non-EEA other migrants, medium stay | 1.18 (1.02-1.36)* |
| non-EEA other migrants, long stay | 1.21 (1.09-1.34)*** |
| Sex |  |
| Men | 1 |
| Women | 1.57 (1.53-1.61)*** |
|  |  |
| Sex*Refugees, short-medium stay | 0.68 (0.57-0.82)*** |
| Sex*Refugees, long stay | 0.65 (0.52-0.82)** |
| Sex*EEA other migrants, short stay | 0.89 (0.74-1.04) |
| Sex*EEA other migrants, medium stay | 0.82 (0.68-0.98)* |
| Sex*EEA other migrants, long stay | 1.08 (0.89-1.31) |
| Sex*non-EEA other migrants, short stay | 0.78 (0.60-1.01)^ |
| Sex*non-EEA other migrants, medium stay | 0.92 (0.77-1.10) |
| Sex*non-EEA other migrants, long stay | 0.88 (0.76.1.02)^ |
| Number of observations=146 785 | |
| ^1^Adjusted for age group, marital status, educational level, income level, number of OPMH consultations and year of first consultation  *p<0.05, **p<0.01, ***p<0.001 | |
